# Measuring context that matters: validation of the modular Tele-QoL patient-reported outcome and experience measure

**DOI:** 10.1007/s11136-023-03469-z

**Published:** 2023-07-17

**Authors:** Klara Greffin, Holger Muehlan, Neeltje van den Berg, Wolfgang Hoffmann, Oliver Ritter, Michael Oeff, Sven Speerfork, Georg Schomerus, Silke Schmidt

**Affiliations:** 1 Department of Psychology, Chair of Health and Prevention, University of Greifswald, Greifswald, Germany; 2 Section Epidemiology of Health Care and Community Health, Institute for Community Medicine, University Medicine Greifswald, Greifswald, Germany; 3 Department of Cardiology, Nephrology and Pulmonology, Campus Clinic Brandenburg, Brandenburg Medical School Theodor Fontane, Brandenburg an der Havel, Germany; 4 Brandenburg City Hospital, Brandenburg an der Havel, Germany; 5 Clinic of Psychiatry and Psychotherapy, University of Leipzig Medical Center, Leipzig, Germany

**Keywords:** Patient-reported outcome measure, Quality of life, Digital health, Telemedicine, Telehealth, Validation

## Abstract

**Purpose:**

A setting-sensitive instrument for assessing Quality of Life (QoL) in Telemedicine (TM) was unavailable. To close this gap, a content-valid “add-on” measure was developed. In parallel, a brief index was derived featuring six items that summarise the main content of the multidimensional assessment. After pre- and pilot-testing, the psychometric performance of the final measures was investigated in an independent validation study.

**Methods:**

The questionnaires were applied along with other standardised instruments of similar concepts as well as associated, yet disparate concepts for validation purposes. The sample consisted of patients with depression or heart failure, with or without TM (*n* = 200). Data analyses were aimed at calculating descriptive statistics and testing the psychometric performance on item, scale, and instrument level, including different types of validity and reliability.

**Results:**

The proposed factor structure of the multidimensional Tele-QoL measure has been confirmed. Reliability coefficients for internal consistency, split-half, and test-retest reliability of the subscales and index reached sufficient values. The Tele-QoL subscales and the index demonstrated Rasch scalability. Validity of both instruments can be assumed. Evidence for discriminant construct validity was provided. Known-groups validity was indicated by respective score differences for various classes of disease severity.

**Conclusion:**

Both measures show convincing psychometric properties. The final multidimensional Tele-QoL assessment consists of six outcome scales and two impact scales assessing (un-)intended effects of TM on QoL. In addition, the Tele-QoL index provides a short alternative for outcome assessment. The Tele-QoL measures can be used as complementary modules to existing QoL instruments capturing healthcare-related aspects of QoL from the patients’ perspective.

**Supplementary Information:**

The online version contains supplementary material available at 10.1007/s11136-023-03469-z.

## Background

Existing health-related or disease-specific quality of life (QoL) questionnaires assess the patient-reported impact of diseases or treatments on the concept [1, 2]. Any aspects related to the context of healthcare, that might influence QoL beyond treatment, were hardly considered so far [3–5]. As part of the digitalization of healthcare, medical procedures and therapeutic treatment strategies are made available within the context of telemedicine (TM; [6]). Furthermore, additional health services are provided through innovative solutions, like telemonitoring [7–11]. This digital transformation has led to a change in healthcare contexts which is widely neglected in TM evaluations [12]. In an extensive review including 293 TM studies [4, 5], results indicated that TM-sensitive instruments were used in only about 5% of the articles included. Moreover, these instruments were only available for a limited range of concepts, as the majority was solely directed to assess satisfaction [13]. Thus, TM-specific aspects of care are not sufficiently covered by existing instruments, yet. Moreover, even though QoL is frequently considered as a core patient-reported outcome [14] in TM [3, 15, 16], there is no QoL instrument available for telehealth in particular. For this reason, we emphasize that more attention should be paid to contextual factors of healthcare, their influence on patients' experiences and health outcomes [12, 17, 18].

The aim of the “Tele-QoL” project was to close this gap by developing a suitable “add-on” QoL instrument to enable a setting-sensitive evaluation of TM applications [19]. As such, this modular questionnaire shall assess QoL of patients with chronic conditions or mental illnesses in the context of TM care. This validation paper aims to document the psychometric performance of the Tele-QoL measures in terms of different forms of reliability and validity.

## Methods

### Instrument development

Developing the Tele-QoL instrument was based on current recommendations for patient-reported outcome measures [20, 21] and to some extent inspired by a needs-based approach of QoL assessment [22]. The items of the Tele-QoL questionnaires were directly derived from qualitative interviews and focus groups, and assess various facets of the healthcare-related domain of QoL [12]. After developing the initial version, an expert workshop for external validation (*n* = 6), an online expert survey to test the instrument's content validity (*n* = 15), and a pre-testing of the initial items with a sample of patients (*n* = 32) were conducted. Subsequently, the revised version of the questionnaire was piloted. Therefore, a sample of patients with depression or heart failure with or without TM care (*n* = 200) was recruited. As a result, we identified an appropriate measurement model comprising six factors related to patient-relevant “outcomes” (Information & Education, Perceived Safety & Well-Being, Needs Orientation & Trust, Perceived Control & Monitoring, Patient Relief & Independence, Cooperation & Communication), and two factors related to the unintended “impact” of telehealth on patients (Data Processing & Surveillance, Patient Burden & Limitation) [23]. The Tele-QoL measure aims to assess healthcare-related aspects of QoL in the context of TM applications (version A) or standard care (comparison version B). It is used as an "add-on instrument" to already existing QoL questionnaires. The target group of the Tele-QoL are patients aged 18 years and older who receive TM care (version A). It is irrelevant whether the patients are being treated for chronic physical or mental illnesses. For now, the Tele-QoL instruments are available in German in form of a short index or full version.

The questionnaire opens with a short instruction on the objective and how to carry it out; this is followed by the respective items. In addition, the temporal reference of four weeks is referred to again at the beginning of each page. Patients rate their healthcare-related experiences of the last four weeks using a 4-point Likert scale with the ratings 1 = "Do not agree" to 4 = "Highly agree". The objective of this paper is to document the evaluation of performance and psychometric properties of the modular Tele-QoL instruments, including the multidimensional Tele-QoL measure with six outcome scales and two impact scales as well as the brief Tele-QoL index with six items. The alternative short version represents the main content of the outcome subscales as closely as possible with one item per dimension, excluding the content of the impact dimensions.

### Data sample

For the validation study, patients with chronic heart failure or depression (*n* = 200), with (Tele-QoL version A) or without (Tele-QoL version B) TM care were recruited. The recruitment was implemented in several hospitals of the project's consortium partners (Brandenburg an der Havel, Greifswald, Leipzig) as well as at ambulatory healthcare facilities, all located in Northeastern Germany. In addition to the disease and treatment criteria mentioned above, a minimum age of 18 was an inclusion criterion; cognitive impairment and severe cognitive comorbidities as well as non-proficient knowledge of the German language were considered as exclusion criteria [19].

Treatment providers or study nurses in the recruitment centers informed interested patients according to pre-defined criteria in person or via telephone about purpose of the study, voluntariness, dropout options, and compensations. In addition, the patients received the information in written form along with phone and e-mail contact details of the recruiting centers and the scientific research assistant. After the patients had given informed consent for the study, the questionnaires were handed out with the request to fill them in. Personal codes were generated for a pseudonymous assignment of the follow-up survey, that was scheduled four weeks later. Personal assistance during the completion of the survey was available upon request. Completed questionnaires were mailed or dropped off in a prepaid envelope. After the questionnaires have been received, data were entered into an Excel spreadsheet and stored on a secured file server. Finally, the original questionnaires were stored in lockable cabinets.

### Applied measures

Whereas all measures were applied to the first wave of the validation study, only some of them were also used within the second wave after four weeks to detect test-retest reliability, stability over time, and sensitivity to change. All instruments included in the validation study are described in the study protocol [19]. Therefore, we will provide a short overview, only.

Sociodemographic characteristics were assessed based on recommended demographic standards in Germany [24]. The “Goldman Specific Activity Scale” (original version: [25]) was used to assess severity of heart failure. Participants were asked to rate whether they are able to perform specific daily activities, and were classified according to four Specific Activity Scale Functional Classes (Class I = least burdened; Class IV = most burdened) based on their answers. It was complemented by the “New York Heart Association Classification” (NYHA; original version: [26]; German version [27]:). Based on their answers, the participants were assigned to one of four classes (NYHA 1 = least burdened; NYHA 4 = most burdened). Depressive symptoms were assessed using the “Patient Health Questionnaire 9 (PHQ-9)” [28]. The “SeCu-20” Questionnaire (German original version: [29]) was used to assess perceived security in experiences with TM care. Patient satisfaction was assessed by the “Questionnaire to evaluate patient satisfaction (ZUF-8)“ (original version: [30]; German version: [31]). Additionally, the general item of the “Youth Health Care Measure (YHC-SUN)” [32] was used to assess the general satisfaction with treatment. Patient activation was assessed using the “Patient Activation Measure (PAM13-D)” (original version: [33]; German version: [34]). To assess body-related self-consciousness, the subscale “private” of the “Body-related Self-Consciousness (KSA)” questionnaire (German original version: [35]) was used. From the “Body-related Locus of Control (KLC)” questionnaire (German original version: [36, 37]) for assessing the body-related locus of control, the subscale “health” was used. The “European Health Literacy Survey (HLS-EU-Q6)” (original version in multiple languages: [38]) was used to assess health literacy. In addition, we used a newly adapted version of HLS-EU-Q6 for digital healthcare, referred to as D-HLS-EU-Q6. The”WHO-Five Well-Being Index (WHO-5)” [39, 40] was used to assess QoL of participants with mental health issues. The “Minnesota Living with Heart Failure questionnaire (MLHFQ)” was used to assess QoL of patients with heart failure [41–43]. The “Veterans RAND 12 Item Health Survey (VR-12)” (original version: [44]; German version: [45]) was used to assess the subjective health status of the participants. Health-related QoL was assessed with the “European Quality of Life 5 Dimensions (EQ-5D)” (original version: [46, 47]). The short form of the “World Health Organization Quality of Life (WHOQOL-BREF)” (original version in multiple languages: [48]) was used to assess the general QoL in four different life domains (physical, psychological, social, environmental).

### Data analyses

*Factorial validity* was investigated by conducting a confirmatory factor analysis with maximum likelihood estimations to test the multidimensional measurement model. Amongst the fit indices [49] we inspected the comparative fit index (CFI), Tucker-Lewis index (TLI), and the root mean square error of approximation (RMSEA). *Discriminant validity* was investigated by calculating Pearson correlation coefficients for associations between Tele-QoL scores and various indicators of general, health-related, and disease-specific QoL as well as measures related to the assessment of satisfaction with care, patient activation, and health literacy. All concepts were assumed to be low or moderately associated with the Tele-QoL scores.

Concerning *convergent validity*, we assumed high associations with the subscales of a setting-sensitive measure for patient experiences in TM. Finally, we tested for correlations with further associated constructs, including self-monitoring and locus of control.

To examine *known-groups validity* with respect to different clinical variables known for differences in QoL, standardized effect sizes for differences of two independent means were estimated using Cohen’s d [50]. We expected that patients with stronger disease severity show lower Tele-QoL outcome and higher impact scores.

*Rasch analysis* was used to detect possible misfit on item level. The partial credit model was applied to the data, using Q index statistics and threshold ordering estimation for detecting item misfit [51].

For *reliability* testing, homogeneity of the subscales was investigated by computing Cronbach's alpha coefficient α. Split-half reliability was determined by the correlation between both test-halves. Intraclass correlation coefficient (ICC) was used to estimate test-retest reliability of the Tele-QoL scores.

### Statistical software

Descriptive statistics and item-scaling analysis were performed using the IBM SPSS Version 28.0 [52]. Confirmatory factor analysis was processed using IBM AMOS Version 28 [53]. For Rasch analysis, the WINMIRA software package was used [54].

## Results

### Sample characteristics

In total, *n* = 200 patients aged 19 to 88 years participated in the Tele-QoL validation study (Table 1). Of these, 51.5% (*n* = 103) reported being male, 48.0% (*n* = 96) female, and 0.5% (*n =* 1) non-binary/gender nonconforming, respectively. Patients included were being treated for chronic heart failure (52.0%, *n* = 104) or depression (48.0%, *n* = 96). Sociodemographic characteristics for each patient group are provided in Table 1. Eighty patients participated in the second wave.

**Table 1:** Sociodemographic characteristics of the Tele-QoL validation study sample (*n =* 200)

Characteristics*	Patients with heart failure		Patients with depression	
	Standard care	Telemedical care	Standard care	Telemedical care
*Age group*				
< *35 years*	–	–	22 (48.0%)	7 (13.7%)
*36—50 years*	1 (1.8%)	1 (2.0%)	12 (26.7%)	11 (21.6%)
*51—65 years*	20 (36.4%)	9 (18.4%)	9 (20.0%)	29 (56.9%)
*66—80 years*	23 (41.8%)	26 (53.1%)	2 (4.4%)	4 (7.8%)
> *80 years*	8 (14.5%)	9 (18.4%)	–	–
*Gender*				
Female	18 (32.7%)	16 (32.7%)	27 (60.0%)	35 (68.6%)
Male	37 (67.3%)	33 (67.3%)	17 (37.8%)	16 (31.4%)
Non-binary / gender nonconforming	–	–	1 (2.2%)	–
*Education (Highest Degree)*				
Primary school (8th/9th class)	3 (5.5%)	10 (20.4%)	3 (6.7%)	2 (3.9%)
Secondary School (10th class)	27 (49.1%)	25 (51.0%)	14 (31.1%)	29 (56.9%)
High School (12th/13th class)	15 (27.3%)	9 (18.4%)	26 (57.8%)	15 (29.4%)
Other Degree	7 (12.7%)	2 (4.1%)	2 (4.4%)	2 (3.9%)
No Formal Degree	2 (3.6%)	1 (2.0%)	–	–

*Data referring to frequencies and percentages. Absolute frequencies vary as a function of the amount of missing data for each variable

**Sum of percent value may vary resulting from rounding of single percent rates

There have been patients who did not want to participate in this study, mostly due to a lack of interest or to feeling overwhelmed by the amount of study participation requests they got from the hospitals. A third group was already stressed due to covid-19 and related circumstances, which was also a factor for non-participation. Unfortunately, the number of these individuals was not systematically recorded in the clinics.

### Data

*Factorial validity* was explored by applying confirmatory factor analysis (CFA). We used Maximum-likelihood parameter estimation for testing the model. Despite impaired normal distribution of items, this method can be applied as it is assumed to be robust even if the data violates the assumption of normal distribution. The model did fit the data well (*χ*^*2*^*(df* = 436) = 696.53, *p* < 0.001, *CFI* = *0.9*4, *TLI* = 0.93, *RMSEA* = 0.056 [0.048; 0.064]).

The six "outcome" subscale scores of the multidimensional Tele-QoL instrument correlate with each other with *r* = 0.39-0.81, the two "impact" subscales with *r* = 0.44 (see Table 2). The Tele-QoL index score correlates moderately to highly with all outcome scales of the multidimensional Tele-QoL (*r* = 0.59−0.83), but slightly negatively with both impact scales (*r* = −0.12 and *r* = −0.16).

**Table 2:** Intercorrelations of the Tele-QoL subscale scores (*n =* 200)

Tele-QoL instruments	Number of items	Tel-QoL outcome scales						Tel-QoL impact scales		Tele-QoL index
Multidimensional Tele-QoL measure		Needs Orientation & Trust	Patient Relief & Autonomy	Information & Education	Cooperation & Communication	Perceived Control & Monitoring	Perceived Safety & Well-Being	Data Processing & Surveillance	Patient Burden & Limitation	Total score
Needs Orientation & Trust	4	**–**	**0.44**	**0.50**	**0.40**	**0.52**	**0.51**	−0.24	−0.25	**0.59**
Patient Relief & Autonomy	4	**0.44**	**–**	**0.69**	**0.63**	**0.47**	**0.39**	-0.07	−0.07	**0.63**
Information & Education	4	**0.50**	**0.69**	**–**	**0.80**	**0.63**	**0.49**	−0.12	−0.13	**0.76**
Cooperation & Communication	4	**0.40**	**0.63**	**0.80**	**–**	**0.60**	**0.53**	−0.06	−0.14	**0.73**
Perceived Control & Monitoring	4	**0.52**	**0.47**	**0.63**	**0.60**	**–**	**0.81**	−0.12	−0.28	**0.83**
Perceived Safety & Well-Being	4	**0.51**	**0.39**	**0.49**	**0.53**	**0.81**	–	−0.20	−0.26	**0.74**
Data Processing & Surveillance	4	−0.24	−0.07	−0.12	−0.06	−0.12	−0.20	**–**	**0.44**	−0.12
Patient Burden & Limitation	4	−0.25	−0.07	−0.13	−0.14	−0.28	−0.26	**0.44**	**–**	−0.16
**Tele-QoL index**	6	**0.59**	**0.63**	**0.76**	**0.73**	**0.83**	**0.74**	−0.12	−0.16	–

Interpretation of correlation coefficients: r < 0.50: low; r = 0.50 - 0.70: moderate; r > 0.70: high. In bold print: r > 0.30

*Rasch analysis* (Partial Credit Model) with emphasis on the operational characteristics of the items showed that none of the items in any of the Tele-QoL subscales or the Tele-QoL index displays infit, indicating no substantial deviation from the model. The range of item locations for most of the scales is moderate (< 2 logits), but the effective range carried by threshold distributions along the latent traits varies between > 4 and < 11 logits. Ordering of thresholds is in accordance with the model assumptions for all items in any of the subscales and the index as well (Table 3).

**Table 3:** Rasch analysis and reliabilities of the multidimensional Tele-QoL sub-scales and the Tele-QoL index (*n =* 200)

Tele-QoL instruments	Number of items	Range of item locations	Range of threshold parameters	Non-ordered thresholds	Item fit (Q index)	Internal consistency	Spilt-half reliability	Test–retest reliability ICC (CI) p-value (t-test)	
Multidimensional Tele-QoL measure		(n_min–max_ = 178–185)	(n_min–max_ = 178–185)	(n_min–max_ = 178–185)	(n_min–max_ = 178–185)	(n_min–max_ = 178–185)	(n_min–max_ = 178–185)	(n_min–max_ = 75–78)	(n_min–max_ = 75–78)
Needs Orientation & Trust	4	−0.84 to 0.75	−3.90 to 3.10	–	0.025 to 0.048	0.90	0.89	0.64 (0.48-0.76)	0.18
Patient Relief & Autonomy	4	−0.13 to 0.23	−2.39 to 2.92	–	0.034 to 0.069	0.87	0.83	0.74 (0.62-0.83)	0.23
Information & Education	4	−0.43 to 0.03	−2.34 to 2.47	–	0.030 to 0.092	0.83	0.83	0.73 (0.61-0.82)	0.48
Cooperation & Communication	4	−1.16 to 0.69	−4.35 to 3.49	–	0.028 to 0.082	0.90	0.84	0.66 (0.51-0.77)	0.36
Perceived Control & Monitoring	4	−0.82 to 0.80	−2.28 to 2.24	–	0.038 to 0.071	0.84	0.91	0.72 (0.59-0.81)	0.46
Perceived Safety & Well-Being	4	−1.17 to 0.96	−6.27 to 4.46	–		0.87	0.84	0.70 (0.57-0.80)	0.07
Data Processing & Surveillance	4	−0.95 to 0.88	−3.20 to 3.00	–	0.019 to 0.050	0.93	0.81	72 (0.59-0.81)	0.13
Patient Burden & Limitation	4	−0.84 to 0.84	−2.60 to 5.63	–	0.021 to 0.038	0.95	0.91	0.72 (0.59-0.81)	0.01*
Tele-QoL index	6	−0.26 to 0.74	−2.71 to 3.05	–	0.046 to 0.075	0.90	0.84	0.69 (0.55-0.79)	0.26

*For reliability testing,* the *internal consistency* was calculated using Cronbach's alpha (α) coefficient for all subscales and the index score. For the Tele-QoL index, a value of *α* = 0.90, and for the Tele-QoL subscales values between *α* = 0.84 and 0.95 were obtained. Thus, the internal consistencies for all scales of the Tele-QoL instruments can be judged as very good. All subscales of the Tele-QoL measure as well as the Tele-QoL index also yielded very good values for the *split-half-reliability*, which varied between 0.81 and 0.91*. Test-retest reliability* was determined over a period of approximately four weeks, controlling for the course of the disease. The corresponding intraclass correlation coefficients vary between 0.64 and 0.74 and are thus sufficient to good. T-tests indicate no significant differences between test and retest scores, except for the subscale “Patient Burden & Limitation” (*p* = 0.01). All reliability coefficients for the subscales of the Tele-QoL and the Tele-QoL index are also depicted in Table 3.

Evidence for *known-groups validity* of the Tele-QoL measure is displayed by expected group differences (*d* = *0.0*1 < 0.44) in the Tele-QoL scores for patients with different disease severity (Table 4). With regard to *discriminant construct validity related to QoL* results show low to moderate correlations with different indices of general QoL (WHOQOL-BREF), health-related QoL (EQ-5D, VR-12), disease-specific QoL, and well-being (MLHFQ, WHO-5). These findings indicate a sufficient *divergent validity* of the Tele-QoL instruments, since they capture different aspects of QoL than other instruments assessing already established concepts of QoL (Table 5). Most coefficients for correlations between the six Tele-QoL outcome subscales with scores from other QoL measures are notably higher for those domains related to mental issues (WHOQOL-BREF: Mental/Psychological Domain, VR-12 Mental Health Status) than for domains related to physical issues (WHOQOL-BREF: Physical Domain, VR-12 Physical Health Status). Also, domains related to social or environmental issues show higher correlations than domains related to physical issues, but not as high as the “mental” domains. Correlation coefficients with physical domains of QoL are generally weak or low (*r* = −0.17 to 0.27). Both impact scales of the multidimensional Tele-QoL measure show low negative correlations with almost all QoL scores (*r* = *−0.2*5 to 0.02).

**Table 4:** Known-groups validity of the Tele-QoL subscales scores and Tele-QoL index score (*n =* 200)

Tele-QoL instruments	Patients with heart failure (NYHA = 1 vs. NYHA > 1)		Patients with depression (PHQ < 15 vs. PHQ > 14)	
Multidimensional Tele-QoL measure	Mean difference*	Effect size Cohen’s d	Mean difference*	Effect size Cohen’s d
Needs Orientation & Trust	0.93	**0.28**	0.42	0.16
Patient Relief & Autonomy	0.33	0.12	1.09	**0.41**
Information & Education	0.52	0.17	1.09	**0.38**
Cooperation & Communication	1.21	**0.33**	1.29	**0.41**
Perceived Control & Monitoring	1.51	**0.44**	0.02	0.01
Perceived Safety & Well-Being	0.63	0.19	0.78	**0.33**
Data Processing & Surveillance	-0.79	−**0.27**	−0.46	−0.18
Patient Burden & Limitation	-0.93	−**0.37**	−0.80	**−0.34**
Tele-QoL index	0.84	0.17	0.94	**0.40**

*Tele-QoL index and subscales scores are linear transformed raw means (range: 0–100). In bold print: d >﻿ 0.20

**Table 5:** Intercorrelations of the Tele-QoL scores with subscale scores of other measures for convergent and discriminant validation (n = 200)

Tele-QoL module	(subscales)	Needs Orientation & Trust	Patient Relief & Autonomy	Information & Education	Cooperation & Communication	Perceived Control & Monitoring	Perceived Safety & Well-Being	Data Processing & Surveillance	Patient Burden & Limitation	Tele-QoL index
Items		*4*	*4*	*4*	*4*	*4*	*4*	*4*	*4*	*6*
WHOQOL-BREF	Physical domain	0.10	0.18	0.21	0.27	0.10	0.05	−0.14	−0.17	0.10
	Mental domain	0.15	**0.40**	**0.36**	0.28	0.17	0.09	−0.18	−0.07	0.27
	Social domain	0.17	**0.34**	0.27	0.21	0.23	0.19	−0.16	−0.04	**0.30**
	Environmental domain	0.17	0.29	0.27	0.25	0.26	0.13	−0.25	−0.22	0.29
EQ-5D	Health-related QoL	0.04	0.06	0.09	0.19	0.01	0.04	−0.21	−0.11	0.01
VR-12	Physical health status	0.04	−0.01	0.01	0.12	−0.01	0.05	−0.13	−0.11	−0.02
	Mental health status	0.18	**0.43**	**0.37**	**0.31**	0.20	0.10	−0.15	−0.12	0.26
MLHFQ *	Disease-specific QoL (Heart failure)	0.20	0.26	**0.33**	**0.34**	**0.34**	0.27	−0.14	−0.15	**0.30**
WHO-5**	Disease-specific QoL (depression)	0.14	**0.37**	0.29	**0.36**	0.17	0.15	0.02	−0.04	0.24
YHC-SUN-1	Satisfaction with healthcare	**0.39**	**0.41**	**0.46**	**0.47**	**0.47**	**0.48**	−0.10	−0.12	**0.52**
ZUF-8	Patient satisfaction	**0.46**	**0.48**	**0.56**	**0.58**	**0.59**	**0.56**	−0.19	−0.17	**0.61**
PAM-13-D	Patient activation	0.22	0.28	**0.33**	**0.34**	0.27	0.22	−0.14	−0.01	**0.33**
HLS-EU-Q6	Health literacy	0.16	0.07	0.07	0.21	0.17	0.28	−0.19	−0.19	0.15
D-HLS-EU-Q6	Digital health literacy	0.13	0.05	0.04	0.18	0.10	0.21	−0.18	−0.12	0.08
KSA	Private body-related self-monitoring	0.10	−0.03	−0.01	−0.01	−0.05	0.03	0.11	0.10	0.05
KLC	Health-related locus of control (internal)	0.17	0.01	0.05	0.02	0.06	0.10	0.01	−0.05	0.04
KLC	Health-related locus of control (external)	−0.08	0.01	0.05	−0.03	−0.01	−0.09	0.03	−0.02	−0.05
TBS	Technology acceptance	0.12	0.12	0.13	0.16	−0.03	−0.00	−0.12	−0.05	0.07
	Technology competence	0.12	−0.09	−0.11	0.01	−0.02	0.06	−0.19	−0.18	−0.04
	Technology control	0.02	−0.05	0.04	0.15	0.02	0.02	−0.08	−0.09	−0.00
SeCu***	Technology anxiety	−0.06	−0.02	−0.04	0.05	0.04	0.07	**0.31**	0.20	0.07
	Perceived security	**0.58**	**0.57**	**0.81**	**0.74**	**0.80**	**0.80**	−0.22	−**0.30**	**0.84**
	Perceived autonomy	**0.38**	**0.63**	**0.78**	**0.90**	**0.62**	**0.66**	−0.08	−0.08	**0.82**
	Physician–patient-relationship	**0.36**	**0.59**	**0.70**	**0.66**	**0.72**	**0.71**	−0.08	−0.17	**0.81**

*For patients with chronic heart failure only; **for patients with depression only, ***for patients with telemedical care only. Interpretation of correlation coefficients: r < 0.50: low; r = 0.50-0.70: moderate; r > 0.70: high. In bold print: r ≥ 0.30. *WHOQOL-BREF* World Health Organization Quality of Life measure (short version); *EQ-5D* EuoQol Quality of Life measure; *VR-12* Veterans RAND 12 Item Health Survey (short version); *MLHFQ* Minnesota Living with Heart Failure Questionnaire; *WHO-5* World Health Organization Well-Being Scale; *YHC-SUN-1* Generic single-item measure of satisfaction with healthcare from the Satisfaction, Utilization & Needs Questionnaire (Youth version); *ZUF-8* Patient Satisfaction Scale (8 item version); *PAM-13-D* Patient Activation Measure; *HLS-EU-Q6* Health Literacy Scale HLS-EU (6 item version); *D-HLS-EU-Q6* (Adopted) Digital version of the Health Literacy Scale HLS-EU (6 item version); *KSA* Body-related Self-Awareness Scale; *KLC* Body-related Locus of Control Scale; *TBS* Technology Commitment Scale; *SeCu* Perceived Security in Telemedicine Scale

*Discriminant construct validity related to patients' experiences with healthcare provision* was also investigated using other measures of related concepts assessing satisfaction with healthcare (YHC-SUN), patient satisfaction (ZUF-8) as well as patient activation (PAM13-D). For almost all correlations between Tele-QoL outcome subscale scores and index score, coefficients indicate low to moderate associations (*r* = 0.22–0.61). In addition, discriminant construct validity was also investigated with selected patient experiences covered by the Tele-QoL scales.

Considering “Information & Education”, health-literacy (HLS-EU-Q6) as well as digital health literacy (D-HLS-EU-Q6)﻿ were assessed.﻿ Correlation coefficients indicate very low associations (*r* < *0.10*). With respect to “Perceived Control & Monitoring”, we applied instruments assessing related concepts such as private body-related self-monitoring (KSA) as well as internal and external health-related locus of control (KLC). Again, correlations coefficients also indicate very low associations (*r* < *0.10*).

All six outcome subscales scores of the Tele-QoL instrument and the index score correlate with the three subscales of the SeCu-instrument assessing patient experiences in TM (*r* = 0.36–0.90). This supports the assumption of *convergent validity*. Missing substantial correlations (*r* = −0.06 < 0.07) with the SeCu subscale assessing negative experiences in TM (“Technology Anxiety”) indicate *divergent validity*. Correspondingly, the subscale"Data Processing & Surveillance" of the Tele-QoL instrument shows a low correlation coefficients with “Technology Anxiety” (*r* = 0.31), but both impact subscales correlate slightly negative with the three “positive” SeCu subscales (*r* = −0.30 < −0.08).

## Discussion

### Main results

With the Tele-QoL measures, we provide a modular instrument that assesses the impact of the TM healthcare context on QoL of patients, beyond the effects of the disease and the treatment [12]. This study tested and determined the psychometric properties of the Tele-QoL measures when applied to a sample of German patients with depression or heart failure.

Summarizing the results of this study, the Tele-QoL measures show a promising psychometric performance. Our results confirm the factorial structure of the multidimensional measure. The multidimensional measure suggests that the Tele-QoL captures distinct domains of healthcare-related QoL in a reliable and valid manner. The reliabilities of all subscales and of the index are satisfying, with alpha coefficients and split-half reliabilities being very good and test retest-reliabilities with sufficient to good values. This indicates that the items within each subscale are highly consistent in measuring the intended construct. Considering that each subscale consists of only four items, these results are quite convincing. Also, operational characteristics of the items for all scales were in line with the model assumptions implied by the Rasch model. Furthermore, this validation study suggests that the Tele-QoL measures possess both discriminant and convergent validity. There is reasonable evidence that the concept of healthcare-related QoL and the domains representing this construct in the measurement model are not identical with related constructs and are sufficiently distinguished from each other in terms of the discriminant validity. This implies that these measures effectively distinguish between various aspects of QoL. In addition, data provides evidence bolstering the incremental validity, indicating that the Tele-QoL measures offer unique insights beyond what is already captured by other QoL questionnaires. Moreover, a high level of content validity can be assumed, as the Tele-QoL questionnaires underwent a rigorous development process, drawing from extensive qualitative material, collected in previous studies of the Tele-QoL project [12]. Noteworthy, the observed correlations among the Tele-QoL outcome subscales imply that there are substantial interrelationships among various aspects of healthcare-related QoL in TM contexts, which is in line with our previous qualitative study [12]. Collectively, these findings affirm the robustness and utility of the Tele-QoL measures in assessing healthcare-related QoL.

### How can the Tele-QoL measures benefit the evaluation of TM applications?

According to a modern understanding, the majority of patients are considered active protagonists who no longer want to be treated passively, but also want to make their own contribution to their health [55, 56]. With a long-lasting illness, however, the needs and challenges in everyday life that a patient is confronted with also increase [57]. For this reason, it is the purpose of TM care for long-term illnesses to support patients in the management of their illness and the needs associated with it [58]. In order to assess whether and to what extent TM applications are able to provide this support, appropriate assessments are needed that reflect the patient's perspective [59]. Therefore, the development and implementation of setting-sensitive questionnaires like the Tele-QoL measures are crucial as they allow for a more valid assessment in TM studies. In this way, the healthcare context is included in the evaluation of care components, in addition to the effects of the disease and respective treatment. As a result, the demand for a valid and quantitative summative evaluation of the medical benefit can now be better met [59].

In general, patients using TM will have the opportunity to better represent the impact of TM on their QoL via the Tele-QoL questionnaire. The extended conceptualization of QoL in TM settings may also lead to potential improvements of TM applications and individualized TM care for patients with chronic diseases and mental illnesses.

### Strengths and limitations

The Tele-QoL is developed based on an extensive mixed-methods approach, which is a strength in terms of content validity [20, 21]. Moreover, patients were included in all stages of the development and validation process. Another advantage is the sample composition for validation, consisting of respondents with complementary diseases and forms of treatment. Thus, half of the sample consisted of patients with TM or standard treatment, half of whom were chronically physically or mentally ill. Amongst patients with TM care, half of them were treated with an active TM approach (regular phone calls), the other half were treated with a passive TM application (remote vital monitoring). The aim was to represent all potential user groups and to test whether the questionnaire can be used independently of the disease and the treatment.

However, our validation study also has limitations. First of all, in planning the project, a compromise had to be made between an adequate sample size and the feasibility of data collection. A sample of *n* = 200 is considered fair [60] and is therefore sufficient, but can be expanded. Future evaluation of the psychometric properties should be based on larger samples, including more disease groups and other TM settings. Moreover, other important properties of the measures need to be investigated, such as readability or responsiveness.

To assess test-retest reliability, patients were asked to complete a second questionnaire four weeks after the initial survey. The date for the second questionnaire was written on the instrument. In addition, after completing the second questionnaire, patients were asked to write the current date under the questionnaire’s items. Unfortunately, not all patients did so. Therefore, we cannot be sure in every case that the questionnaires were filled out exactly four weeks later.

The severity of the respective disease, which was used for calculating the known-groups validity, was based on patients’ self-reports, assessed via patient-reported outcome measures. The data may be biased, for example, by how someone feels on a particular day. In addition, the validation was conducted as a questionnaire study in which patients were asked to fill out different questionnaires one after the other. We arranged the order of the questionnaires in such a way that the questions on general health run towards specific health questions in order to cause as little priming as possible. Nevertheless, answering one questionnaire may have an impact on answering subsequent questionnaires.

It remains unclear what effect the SARS-CoV-2 pandemic outbreak had on our sample. The recruiting institutions had the impression that more severely burdened patients were less willing to participate in the study than before the pandemic, but this circumstance was not systematically recorded. Nevertheless, it should be reported that in this context there may have been a selection and nonresponse bias in our sample regarding the severity levels included. Besides, TM was suddenly used as a substitute, not as a complement.

In summary, this instrument development demonstrates that the psychometric properties of the Tele-QoL measures are convincing. However, it only remains the first step towards a fully validated questionnaire [61].

### Conclusion & outlook

The modular Tele-QoL instrument represents a methodologically sound measure to assess QoL in TM settings. It can be used as complementary module to existing QoL instruments to assess healthcare-related aspects of QoL from the patients’ perspective in telehealth contexts. It is an important and necessary contribution to developing, implementing, and evaluating digital health applications. In the future, the Tele-QoL approach will be further adapted so that it can also be used for children and adolescents (new development of a Tele-QoL Kids) as well as in other countries (cultural adaptation and translation) facing similar healthcare challenges.

### Supplementary Information

Below is the link to the electronic supplementary material.

Supplementary file1 (DOCX 24 kb)

## Data Availability

Data of the Tele-QoL study are available upon reasonable request by the corresponding author.

## Declarations

### Research involved in human and animal participant

The authors assert that all procedures contributing to this work comply with the ethical standards of the relevant national and institutional committees on human experimentation and with the Helsinki Declaration of 1975, as revised in 2008.

### Preregistration and study protocol

The study protocol was published in the journal IJERPH "Setting-sensitive conceptualization and assessment of QoL in telemedical care - Study protocol of the Tele-QoL project" [19].
