# Correction to: Measuring context that matters: Validation of the modular Tele-QoL patient-reported outcome and experience measure

**DOI:** 10.1007/s11136-023-03594-9

**Published:** 2024-04-08

**Authors:** Klara Greffin, Holger Muehlan, Neeltje van den Berg, Wolfgang Hoffmann, Oliver Ritter, Michael Oeff, Sven Speerfork, Georg Schomerus, Silke Schmidt

**Affiliations:** 1 Department of Psychology, Chair of Health and Prevention, University of Greifswald, Greifswald, Germany; 2 Section Epidemiology of Health Care and Community Health, Institute for Community Medicine, University Medicine Greifswald, Greifswald, Germany; 3 Department of Cardiology, Nephrology and Pulmonology, Campus Clinic Brandenburg, Brandenburg Medical School Theodor Fontane, Brandenburg an der Havel, Germany; 4 Brandenburg City Hospital, Brandenburg an der Havel, Germany; 5 Clinic of Psychiatry and Psychotherapy, University of Leipzig Medical Center, Leipzig, Germany

## Correction to: Quality of Life Research (2023) 32:3223–3234 10.1007/s11136-023-03469-z

This correction addresses an error present in Table 3 within the manuscript. The error pertains to a formatting issue in the column documenting "Item fit (Q index)" values, in which one cell contains a missing value and selected other values are swapped. This discrepancy seems to have occurred due to format distortions.

**Table 3:** Rasch analysis and reliabilities of the multidimensional Tele-QoL sub-scales and the Tele-QoL index (*n* = 200)

Tele-QoL instruments	Number of items	Range of item locations	Range of threshold parameters	Non-ordered thresholds	Item fit (Q index)	Internal consistency	Spilt-half reliability	Test–retest reliability ICC (CI) p-value (t-test)	
*Multidimensional* *Tele-QoL measure*		*(n *_*min–max*_ = *178–185)*	*(n *_*min–max*_ = *178–185)*	*(n *_*min–max*_ = *178–185)*	*(n *_*min–max*_ = *178–185)*	*(n *_*min–max*_ = *178–185)*	*(n *_*min–max*_ = *178–185)*	*(n *_*min–max*_ = *75–78)*	*(n *_*min–max*_ = *75–78)*
Needs Orientation & Trust	4	− 0.84 to 0.75	− 3.90 to 3.10	–	.025 to .048	.90	.89	0.64 (0.48 –0.76)	0.18
Patient Relief & Autonomy	4	− 0.13 to 0.23	− 2.39 to 2.92	–	.034 to .069	.87	.83	0.74 (0.62–0.83)	0.23
Information & Education	4	− 0.43 to 0.03	− 2.34 to 2.47	–	.038 to .071	.83	.83	0.73 (0.61–0.82)	0.48
Cooperation & Communication	4	− 1.16 to 0.69	− 4.35 to 3.49	–	.019 to .050	.90	.84	0.66 (0.51–0.77)	0.36
Perceived Control & Monitoring	4	− 0.82 to 0.80	− 2.28 to 2.24	–	.030 to .092	.84	.91	0.72 (0.59 –0.81)	0.46
Perceived Safety & Well-Being	4	− 1.17 to 0.96	− 6.27 to 4.46	–	.021 to .038	.87	.84	0.70 (0.57–0.80)	0.07
Data Processing & Surveillance	4	− 0.95 to 0.88	− 3.20 to 3.00	–	.028 to .082	.93	.81	0.72 (0.59–0.81	0.13
Patient Burden & Limitation	4	− 0.84 to 0.84	− 2.60 to 5.63	–	.031 to .094	.95	.91	0.72 (0.59–0.81)	0.01*
***Tele-QoL index***	6	− 0.26 to 0.74	− 2.71 to 3.05	–	.046 to .075	.90	.84	0.69 (0.55–0.79)	0.26

Additionally, due to an omission of the editorial team in the publication process, the item content and Rasch Model parameters were not published in the final version of the manuscript. The following tables present the estimated model parameters and the translated content of the questionnaire. It is important to note that while this version has not undergone formal validation, it is a carefully reviewed translation of the validated German questionnaire (see also a full version including instructions presented at the end of this correction).

**Table A1:** Item parameters of the multidimensional Tele-QoL sub-scales and the Tele-QoL index (n = 200)

**Factor**	**Item content**	**Item location**	**Item threshold parameters**			**Item fit**		
						*Q index*	*Zq *	*p(X>Zq)*
*Tele-QoL index*								
	• I feel safer through telemedicine.	-0.18134	-1.517	-0.935	1.908	0.0561	-0.0663	0.52641
	• I can arrange my everyday life more freely through telemedicine.	0.74307	-0.863	0.042	3.051	0.0619	0.2239	0.41140
	• I am informed about my health through telemedicine check-ups.	-0.19928	-2.718	-0.634	2.154	0.0749	0.1873	0.42573
	• I am constantly accompanied in managing my condition through telemedicine.	-0.17449	-1.698	-0.607	1.781	0.0488	-0.1542	0.56128
	• I perceive my telemedical treatment as tailored precisely to me.	0.27064	-0.930	-0.465	2.207	0.0460	-0.1397	0.55556
	• I am informed about my telemedical treatment.	-0.25859	-1.440	-0.588	1.252	0.0533	-0.0450	0.51795
*Information & Education*								
	• I was explained how my telemedical treatment works.	- 0.03625	- 2.007	- 0.519	2.417	0.0380	- 0.2222	0.58793
	• I have understood what happens to me as part of the telemedical treatment.	0.02690	- 1.635	- 0.552	2.267	0.0460	- 0.0975	0.53885
	• I am informed about the limitations of my telemedical treatment.	- 0.43306	- 2.348	-0.740	1.788	0.0458	- 0.1403	0.55578
	• As part of my telemedical treatment, I receive exactly the information that is important for me.	0.44241	- 1.250	0.112	2.466	0.0711	0.4683	0.31979
*Perceived Control & Monitoring*								
	• Through telemedical check-ups, I pay more attention to the signals of my body.	0.31879	- 1.273	0.255	1.974	0.0915	0.3629	0.35833
	• Through telemedicine, I know how to interpret my symptoms.	0.80201	- 0.586	0.757	2.235	0.0624	0.1352	0.44622
	• Through telemedicine, I can assess when I should seek additional medical assistance.	- 0.82362	- 2.276	- 0.924	0.729	0.0304	- 0.3566	0.63929
	• The telemedical measures give me a sense of control.	0.22064	- 2.108	- 0.243	3.103	0.0519	- 0.1231	0.54899
*Data Processing & Surveillance*								
	• I am worried that my health data could be misused.	-0.30033	-2.532	0.594	1.037	0.0350	- 0.1904	0.57549
	• I am concerned that unauthorized individuals might access my health data.	-0.95961	-3.196	-0.310	0.627	0.0315	0.0792	0.46842
	• I am afraid that my privacy could be compromised through telemedical check-ups.	0.37543	-1.799	0.644	2.281	0.0280	- 0.6156	0.73094
	• I feel like I am being controlled by the telemedical treatment.	0.88451	-1.011	0.661	3.004	0.0821	0.8083	0.20947
*Perceived Safety & Well-Being*								
	• Through telemedicine, I also feel well taken care of at home.	- 0.03698	- 3.320	- 0.142	3.351	0.0312	0.0198	0.49209
	• Thanks to telemedical treatment, I feel more internally calm.	- 1.17248	- 6.270	- 0.735	3.487	0.0212	- 0.1087	0.54329
	• Thanks to telemedicine, I feel safer in dealing with my condition.	0.24803	- 2.617	- 0.042	3.403	0.0383	0.1131	0.45498
	• The telemedical recording of my health data gives me a sense of safety.	0.96143	- 1.903	0.324	4.463	0.0241	0.0241	0.50975
*Patient Relief & Autonomy*								
	• I feel supported in my everyday life through telemedical measures.	0.23182	-1.422	-0.508	2.625	0.0688	0.2820	0.38897
	• I can be more active in my daily life through telemedicine.	-0.07490	-2.394	-0.755	2.924	0.0342	-0.2713	0.60692
	• Thanks to telemedical treatment, I am more independent in my everyday life.	-0.12844	-1.472	-0.943	2.029	0.0502	-0.1059	0.54218
	• Telemedicine helps me cope better with difficult situations.	-0.02847	-1.892	-0.427	2.234	0.0598	0.0949	0.46220
*Patient Burden & Limitation*								
	• Using telemedicine involves a high level of bureaucratic effort for me.	- 0.84351	- 2.588	- 0.480	0.537	0.0943	1.0589	0.14483
	• Using telemedicine burdens me.	0.83916	- 2.600	- 0.511	5.628	0.0314	- 0.6126	0.72994
	• Using telemedicine overwhelms me.	0.21057	- 1.795	0.025	2.401	0.0677	- 0.0417	0.51662
	• Using telemedicine restricts me in my everyday life.	- 0.20622	- 1.910	0.194	1.098	0.0428	- 0.5023	0.69226
*Needs Orientation & Trust*								
	• My personal needs are taken into account during the telemedical treatment.	- 0.13150	- 1.906	- 0.773	2.284	0.0285	-0.1196	0.54758
	• The telemedical treatment is precisely tailored to my needs.	- 0.83569	- 3.908	- 0.719	2.120	0.0247	-0.1724	0.56845
	• I have confidence in my telemedical treatment measures.	0.74655	- 0.893	0.487	2.645	0.0454	0.1763	0.43004
	• I can rely on my telemedical treatment.	0.22064	- 2.108	- 0.243	3.103	0.0476	0.1196	0.45240
*Communication & Cooperation*								
	• I have the opportunity to receive information about my treatment even at short notice.	- 1.16153	- 4.354	- 1.297	2.166	0.0501	0.2433	0.40389
	• I feel understood by my telemedical contact person.	0.34593	- 1.985	- 0.092	3.114	0.0194	- 0.4215	0.66331
	• My telemedical contact person and I communicate on an equal footing.	0.68603	- 1.375	- 0.060	3.494	0.0298	- 0.0816	0.53251
	• My telemedical contact person motivates me to actually implement agreements.	0.12958	- 2.114	- 0.727	3.230	0.0453	0.2465	0.40264

### Tele-QoL

#### English version

### Instructions

For some time now, you have been undergoing telemedical treatment due to a chronic physical or mental condition. We would like to ask you about this in the following. Please answer each of the questions by choosing the option that best applies to you. When answering the questions, please consider how you have been doing on average in the past 4 weeks.

### Index-A

- I feel safer through telemedicine.
- I can arrange my everyday life more freely through telemedicine.
- I am informed about my health through telemedicine check-ups.
- I am constantly accompanied in managing my condition through telemedicine.
- I perceive my telemedical treatment as tailored precisely to me.
- I am informed about my telemedical treatment.

### Tele-QoL-A

1. I was explained how my telemedical treatment works.
2. I have understood what happens to me as part of the telemedical treatment.
3. I am informed about the limitations of my telemedical treatment.
4. As part of my telemedical treatment, I receive exactly the information that is important for me.
5. Through telemedical check-ups, I pay more attention to the signals of my body.
6. Through telemedicine, I know how to interpret my symptoms.
7. Through telemedicine, I can assess when I should seek additional medical assistance.
8. The telemedical measures give me a sense of control.
9. I am worried that my health data could be misused.
10. I am concerned that unauthorized individuals might access my health data.
11. I am afraid that my privacy could be compromised through telemedical check-ups.
12. I feel like I am being controlled by the telemedical treatment.
13. Through telemedicine, I also feel well taken care of at home.
14. Thanks to telemedical treatment, I feel more internally calm.
15. Thanks to telemedicine, I feel safer in dealing with my condition.
16. The telemedical recording of my health data gives me a sense of safety.
17. I feel supported in my everyday life through telemedical measures.
18. I can be more active in my daily life through telemedicine.
19. Thanks to telemedical treatment, I am more independent in my everyday life.
20. Telemedicine helps me cope better with difficult situations.
21. Using telemedicine involves a high level of bureaucratic effort for me.
22. Using telemedicine burdens me.
23. Using telemedicine overwhelms me.
24. Using telemedicine restricts me in my everyday life.
25. My personal needs are taken into account during the telemedical treatment.
26. The telemedical treatment is precisely tailored to my needs.
27. I have confidence in my telemedical treatment measures.
28. I can rely on my telemedical treatment.
29. I have the opportunity to receive information about my treatment even at short notice.
30. I feel understood by my telemedical contact person.
31. My telemedical contact person and I communicate on an equal footing.
32. My telemedical contact person motivates me to actually implement agreements.

