# Supplementary material for: Measuring context that matters: validation of the modular Tele-QoL patient-reported outcome and experience measure
Source: Qual Life Res. 2023 Jul 17;32(11):3223–34. doi: 10.1007/s11136-023-03469-z (PMC10522723; doi:10.1007/s11136-023-03469-z)
Supplement: Supplementary file 1 — Supplementary file1 (DOCX 24 kb) [file 11136_2023_3469_MOESM1_ESM.docx]

**Supplementary Material**

**Table S1**

*Standardized Regression Weights (CFA)*

| **Latent Factor** | **Item** | **Estimate** |
| --- | --- | --- |
| - *Needs Orientation & Trust* |  |  |
|  | Item 01 | .867 |
|  | Item 02 | .881 |
|  | Item 03 | .853 |
|  | Item 04 | .870 |
| - *Patient Relief & Autonomy* |  |  |
|  | Item 05 | .777 |
|  | Item 06 | .916 |
|  | Item 07 | .861 |
|  | Item 08 | .809 |
| - *Information & Education* |  |  |
|  | Item 09 | .900 |
|  | Item 10 | .888 |
|  | Item 11 | .785 |
|  | Item 12 | .823 |
| - *Cooperation & Communication* |  |  |
|  | Item 13 | .638 |
|  | Item 14 | .725 |
|  | Item 15 | .897 |
|  | Item 16 | .832 |
| - *Perceived Control & Monitoring* |  |  |
|  | Item 17 | .698 |
|  | Item 18 | .815 |
|  | Item 19 | .786 |
|  | Item 20 | .724 |
| - *Perceived Safety & Well-Being* |  |  |
|  | Item 21 | .858 |
|  | Item 22 | .832 |
|  | Item 23 | .816 |
|  | Item 24 | .651 |
| - *Data Processing & Surveillance* |  |  |
|  | Item 25 | .866 |
|  | Item 26 | .812 |
|  | Item 27 | .892 |
|  | Item 28 | .732 |
| - *Patient Burden & Limitation* |  |  |
|  | Item 29 | .629 |
|  | Item 30 | .842 |
|  | Item 31 | .784 |
|  | Item 32 | .852 |
